# Selective Consecutive Determination of Desloratadine and Montelukast Sodium in Their Pure and Binary Dosage Form Based on Pencil Graphite Electrochemical Sensors

**DOI:** 10.1155/2021/5540907

**Published:** 2021-05-04

**Authors:** Amir Alhaj Sakur, Dania Nashed, Imad Noureldin

**Affiliations:** Analytical and Food Chemistry Department, Faculty of Pharmacy Aleppo University, Aleppo, Syria

## Abstract

In this study, we present a new, green electrochemical method for potentiometric estimation of desloratadine and montelukast sodium in their pure and binary dosage form. For that, three pencil graphite sensors were fabricated; the first one was prepared to analyse desloratadine drug (DES) by coating the graphite bar with the coating membrane, which comprises the ion pair of desloratadine and ammonium reineckate reagent (RNK), the polymer poly vinyl chloride (PVC), and the plasticizers dibutyl phthalate (DBP). The second one, which was used to analyse montelukast (MON), was constructed by using the ion pair of cadmium chloride reagent (Cd.) with montelukast and the same earlier named polymer and plasticizer. As a trial to analyse both of the drugs by the same sensor consecutively, we have constructed a combined pencil graphite electrode, which contains the two earlier suggested ion pairs, that is, we can use this electrode to selectively analyse for each drug. The proposed electrodes were effectively used for analysis of DES and MON as a single dosage form and as combined pharmaceutical preparation, without any need for prior separation that was performed depending on the difference in the efficient pH range for each sensor. The proposed sensors exhibited a Nernstian equation slopes of −30.11, 27.70, (−29.16, 29.79) mv. decade^−1^ in the linearity range 5.00 × 10^−5^−1.00 × 10^−2^ and 1.00 × 10^−5^ − 1.00 × 10^−2^ M, respectively. The sensors exhibit high sensitivity according to LOD values ((0.036–0.018) − (0.025-0.026) *µ*M), respectively, and important selectivity toward the studied drugs in presence of interfering ions and excipients. The optimum circumstances were studied, and the method was validated by application of ICH rules. Finally, the method was compared with a documented method, and the required statistical values were calculated.

## 1. Introduction

In the last few years, there has been an urgent need to design new combined dosage forms to enhance the efficacy or reduce the side effects of the drugs. That demands develop new analytical techniques, which can analyse these combinations in a fast, simple, precise way [1].

**Figure 1:**
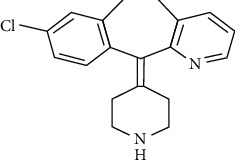
Desloratadine structure.

Desloratadine (DES), 8-chloro-6,11-dihydro-11-(4-piperidylidene)-5H benzo [2, 3] cyclohepta [1,2-b] pyridine [4] (Figure 1), is a nonsedating antihistamine used in the symptomatic release of allergic conditions such as rhinitis and urticaria [5]. The literature review revealed that several methods are available for the determination of DES individually including HPLC [2, 6], spectrophotometric [3, 7], and voltammetric [8]. However, there is not any potentiometric method to determine DES before this study.

**Figure 2:**
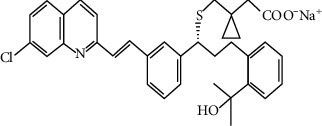
Montelukast sodium structure.

Montelukast sodium is (1-(1R)-1-[3-[(1E)-2-(7-chloro-2-quinolinyl) ethenyl] phenyl]-3-[2-(1-hydroxy-1-methylethyl) phenyl]-propyl] thio] methyl) cyclopropane acetic acid [4] (Figure 2). It is a cysteinyl leukotriene receptor antagonist and used for treatment of asthma [9]. Some research studies were reported to determine MON as individual drug such as HPLC [10, 11], spectrophotometric [12, 13], and potentiometric [14–16].

The combination of desloratadine and montelukast sodium significantly improved nasal symptoms, and it is used for treatment of asthma, allergies, and chronic urticaria [17]. Some reported methods have been used for the simultaneous determination of desloratadine and montelukast such as HPLC [18, 19] and spectrophotometry [20, 21]. However, no electrochemical method is mentioned in the literature to determine DES and MON as a combined form. This promoted us to develop a new simple, sensitive, selective, rapid, and green potentiometric technique using a pencil graphite sensor for the analysis of the two drugs in their coformulated tablets. Electrochemical methods have a lot of advantages compared with the other analytical methods because of their selectivity, sensitivity, simplicity, and the wide linear range which allow the analyst to determine drugs and ions in small concentrations [22–26]. Among all the electrochemical techniques, pencil graphite electrodes have many features such as the small size, their fast response time, and the long lifetime [14, 27] compared to those classical ion-selective electrodes [28–31]. The main purpose of the study is to draw attention to the ability of electrochemical methods for quantitative estimation of drugs in their combination forms competitively with the other common analytical techniques used to determine combination forms such as HPLC and spectrophotometric methods, which were considered as complex, time-consuming, and organic solvent-consuming methods.

## 2. Materials and Methods

### 2.1. Chemicals

All used solvents and materials were analytical class; high pure desloratadine and montelukast sodium, polyvinyl chloride (PVC), tetrahydrofuran (THF), and dibutyl phthalate (DBP) were purchased from Sigma-Aldrich, Germany.

Ammonium reineckate and cadmium chloride were purchased from BDH Chemicals, England.

Bidistilled water was used to prepare solutions.

### 2.2. Apparatus

  Radiometer analytical—ion check 10 pH/mv meter (Cedex, France)
  Crison pH meter model Glp21/EU (Spain)
  Ultrasonic bath—Powersonic 405 (Korea)
  Sartorius balance model 2474 (Germany)

### 2.3. Standard Solutions

#### 2.3.1. Standard Solution of DES

A stock solution of DES (1.00 × 10^−2^ M) was made by dissolving exact weight equivalent to 0.155 g of drug powder in a 50 ml volumetric flask using bidistilled water as a solvent; then, a series of working solutions, their concentrations varying 1.00 × 10^−7^ − 1.00 × 10^−3^ M, were prepared by continuing dilutions from the first stock solution.

#### 2.3.2. Standard Solution of MON

A stock solution of MON (1.00 × 10^−2^ M) was made by dissolving exact weight equivalent to 0.304 g of drug powder in a 50 ml volumetric flask using bidistilled water as a solvent; then, a series of working solutions, their concentrations varying 1.00 × 10^−7^ − 1.00 × 10^−3^ M, were prepared.

### 2.4. Pharmaceutical Formulations

  Aerius (5 mg F.C. Tab). Each tablet is claimed to contain 5 mg of DES, and it was manufactured by Unipharma pharmaceutical company (Syria).
  Azmalir (10 mg F.C. Tab). Each tablet is claimed to contain 10 mg of MON, and it was manufactured by Unipharma pharmaceutical company (Syria).
  Desolate-M Tab. Each tablet contains 5 mg DES and 10 mg MON, and it was manufactured by Archicare Limited (India).

### 2.5. Procedure

#### 2.5.1. Fabrication of the Sensors

The first step to fabricate the sensor is to prepare the ion pairs of drugs and reagents. For the DES graphite sensor, 1 mmol of DES was mixed with 1 mmol of ammonium reineckate, and a pink precipitate was formed. For the MON graphite sensor, 1 mmol of MON was mixed by 1 mmol of cadmium chloride, and a yellow precipitate was formed. Then, the precipitates were filtered and washed several times by bidistilled water to be used later as an electroactive material in the coating solutions for the two electrodes separately [16].

The second step is to prepare the coating solutions, 0.6 g of PVC with 1.2 g of DBP; then, we added 0.2 g of IP (DES and RNK in case of sensor 1 and MON and Cd. in case of sensor 2) for the combined sensor, 0.48 g PVC with 0.96 g DBP; then, we added 0.2 g of each IP (DES.RNK and MON.Cd.). Then, all the components were dissolved in a small volume of THF. The last step is to fabricate the pencil-coated graphite electrodes, performed by immersing the end of a graphite rod (2 mm in diameter) in the previous coating solutions several times to get a suitable thickness of polymeric film that was required [32]. Each electrode was activated before the measurement of the potential by dipping it in 1.00 × 10^−3^ M of drug solution for 24 hrs.

#### 2.5.2. Sensors Calibration

The fabricated sensors were immersed in junction with the Ag/AgCl reference electrode in standard series solutions of DES or MON (1 × 10^−6^–1 × 10^−2^ M) separately. The potential produced by the suggested electrodes was read for each concentration. Calibration graphs were constructed related to the electrode potential values versus the negative logarithmic value of the drug concentration [33].

#### 2.5.3. Potential Measurement Conditions of the Proposed Sensors

We have studied some of the conditions that may have an important effect on the electrode potential; these conditions are the effect of components percentage, the effect of pH and temperature, response time, and the selectivity of the electrode in existence of several obstructive ions and excipients.

#### 2.5.4. Preparation of the Sample's Solutions

The proposed sensors were used for the determination of desloratadine and montelukast sodium in some pharmaceutical preparations as single and combined dosage forms. Twenty tablets of each medication were softly powdered; precise weight proportionate to one tablet was taken, dissolved, and sonicated in the ultrasonic bath for 5 minutes and filtered. An exact volume was taken from the filtrate and diluted to 25 ml to get 10^−4^ M of drug solution.

## 3. Results and Discussion

In this study, we determine each of DES and MON in their pure and combined form, depending on the idea that DES acts as a cation in which it makes up an ion pair with reineckate anion and MON acts as an anion in which it makes up an ion pair with cadmium cation. We can determine each drug using its proposed sensor without any obstruction from the other drug potentials, depending on the difference in the active pH range for each sensor, so that we were able to determine each of these drugs in their binary mixture by applying an accurate, precise, sensitive method, which presents a new potentiometric method to analyse DES and MON combination, instead of other sophisticated analytical methods reported to analyse this combination.

### 3.1. Performance Characteristics of the Developed Sensors

The constructed sensors in conjunction with the Ag/Ag Cl reference electrode were used for the direct determination of DES and MON in their standard series solutions, and their concentrations range 1 × 10^−6^ − 1 × 10^−2^ M. Calibration slope for each sensor was measured from day to day and found to be almost stable over a period of 63 days for the DES graphite sensor and of 49 days for the MON graphite sensor. The performance characteristics of the proposed graphite sensors are given in Table 1.

### 3.2. Effect of the Percentage of Coating Solution's Components

The coating solution, which was used as a coating film covering the pencil graphite electrode, consists of the polymer (PVC), plasticizer (DBP), and the electroactive material (IP_S_). We have tried different percentages of these components to get the best sensor's characteristics, as given in Table 2.

### 3.3. Effect of pH and Temperature

**Figure 3:**
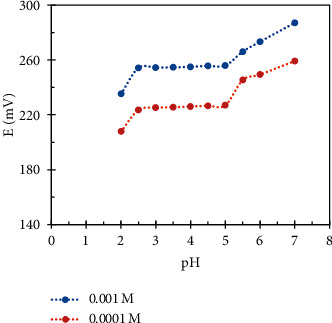
Effect of pH on potentiometric response for the DES.RNK sensor.

**Figure 4:**
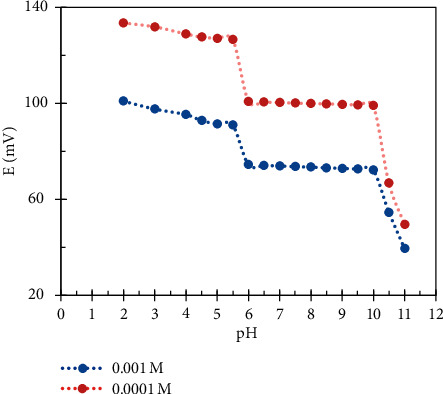
Effect of pH on potentiometric response for the MON.Cd. sensor.

To determine the active pH range for each sensor, two concentrations (1 × 10^−4^ M and 1 × 10^−3^ M) of each drug were studied separately over pH range 2–11 using NaOH and HCl 0.1 M to adjust the pH value. The potential was measured and plotted versus pH values for each drug using its proposed sensor. As shown in Figures 3 and 4, we have found that the effective pH range was 2.5–5 for the DES.RNK sensor; at PH values more than 5, the sensor's potential was unstable and unbalanced. For MON.Cd. sensor, the effective pH range was 6–10; at pH value less than 6, the drug transferred to the unionized form which led to instability in the response.

**Figure 5:**
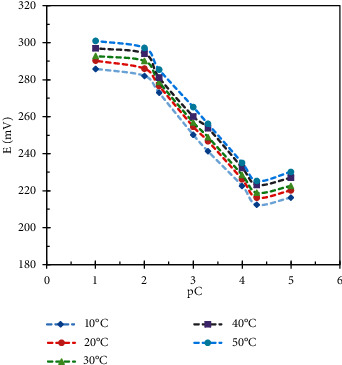
Effect of temperature on DES.RNK sensor response.

**Figure 6:**
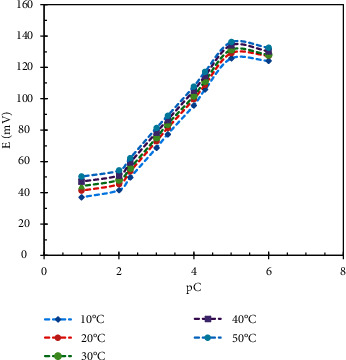
Effect of temperature on MON.Cd. sensor response.

The influence of the temperature on the sensors' response was studied in the range of temperatures 10–50°C. We have found a gradual increase in electrodes' potential as the temperature increases. Calibration graphs at each temperature value were plotted, and we found that the slope values remain almost stable over temperatures range 10–50°C, which indicates the thermal stability of the constructed electrodes up to 50°C (Figures 5 and 6). At temperature values more than 50°C, an obvious decrease in the slope value was found and that maybe due to the instability of the ion pairs in high temperature values.

### 3.4. Selectivity

The selectivity of the constructed sensors towards the drug's ion was studied using the matched potential method [34]. Furthermore, we studied the selectivity of the DES.RNK sensor toward desloratadine in presence of montelukast drug and the selectivity of MON.Cd. toward MON in presence of DES drug, and the results exhibit high selectivity as given in Table 3.

### 3.5. Response Time and Reversibility

**Figure 7:**
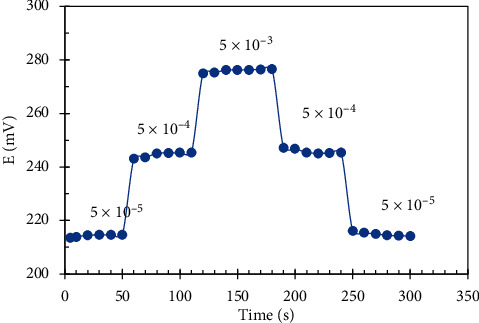
Dynamic response time and reversibility of DES.RNK electrode.

**Figure 8:**
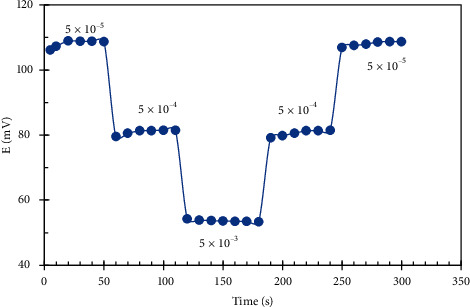
Dynamic response time and reversibility of MON.Cd. electrode.

The response time of the constructed pencil graphite sensors was estimated by immersing the electrodes in the series of drug solutions, each has a 10-fold increase in concentrations 5 × 10^−5^, 5 × 10^−4^, and 5 × 10^−3^ M, and measuring the average time needed to reach a steady potential within ±0.1 mV [33]. As noticed from Figures 7 and 8, the time required to get a constant potential of the final equilibrium value is less than 20 s.

To estimate the reversibility of the proposed graphite sensors, we recorded the potential response of 3 drug concentrations which are within the linearity range (5 × 10^−5^, 5 × 10^−4^, and 5 × 10^−3^ M) from the lowest concentration to the highest and vice versa [35]. We found that the response was reversible as seen in Figures 7 and 8, but we noticed that time required to achieve the equilibrium in potential response was much higher in case of changing concentrations from the highest to the lowest. [33].

### 3.6. Method Validation

We have validated the proposed method, according to the ICH rules [36] as following.

#### 3.6.1. Linearity

**Figure 9:**
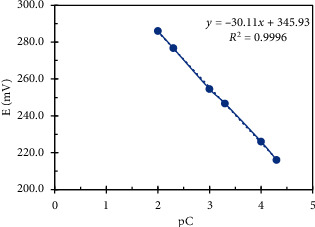
Potentiometric linearity profile of DES.RNK graphite sensor.

**Figure 10:**
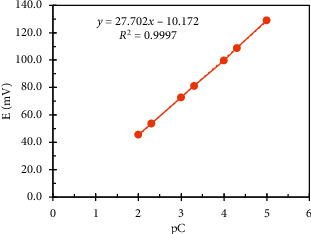
Potentiometric linearity profile of MON.Cd. graphite sensor.

To estimate the linearity range of the constructed sensors, the potentials of a standard series consisting of 10 concentrations ranging between 1 × 10^−6^ M and  1 × 10^−1^ M were measured to get the regression equation for each sensor; the linearity range was found to be 5 × 10^−5^ − 1 × 10^−2^ M and 1 × 10^−5^ − 1 × 10^−2^ M for the DES sensor and MON sensor respectively, as shown in Figures 9 and 10. When we used the combined sensor, we noticed a wider linearity range for both drugs 1 × 10^−5^ − 1 × 10^−2^ M and 5 × 10^−6^ − 1 × 10^−2^ M for DES and MON, respectively, that maybe due to the higher percentage of the electroactive material in membrane composition.

#### 3.6.2. Accuracy and Precision

The method has been proven to be accurate by calculating the recovery values for 3 concentrations, which were chosen within the linearity range by direct potentiometric measurements. Each concentration was measured in triplicate, and the average recovery values were 100.95% and 99.60% for sensor 1 and sensor 2, respectively, that approved the accuracy of the proposed method.

The precision of the method was verified at two levels (repeatability and intermediate precision) and was performed by triplicate determination for 3 concentrations which were measured on the same day for the interday level and on 3 different days for the intraday level. The values of RSD, which were <2%, indicate the precision of the method, as given in Table 1.

#### 3.6.3. Specificity

The specificity of the proposed method was confirmed by preparing laboratory mixtures of desloratadine and montelukast sodium at different ratios. The recovery for each drug was determined by its proposed sensor in presence of the other drugs. The recovery values which are given in Table 4 indicate the specificity of the method.

#### 3.6.4. Pharmaceutical Applications

The proposed method was successfully applied to determine desloratadine and montelukast sodium in their pharmaceutical preparations as single dosage and as a combined dosage form (desolate-M), without any separation depending on the effective pH range for each electrode. The recovery outcomes which are given in Table 5 reveal the ability of the proposed sensors to be used for direct potentiometric determination of drugs in its pharmaceutical forms without any interference of the excipients. The statistical tests (*t* test and *F* test) were applied to confirm that the results which we got do not differ from the results which were reported in a reference HPLC method [19] that denote the accuracy and precision of the proposed method.

## 4. Conclusions

Summing up the results, it can be concluded that the proposed pencil graphite sensors can be used as a selective, sensitive, and validated analytical technique for the potentiometric determination of desloratadine and montelukast in their combined dosage form without any separation step. This research was the first trial for electrochemical determination of this combination. The proposed method could compete with the complicated methods which were reported to determine this combination, being simpler, faster, ecofriendly, moreover the wide linear range and long lifetime of the electrodes that save time and the efforts of the analysis process.

## Figures and Tables

**Table 1:** The performance characteristics of the proposed described sensors.

Parameter	DES.RNK graphite sensor	MON.Cd. graphite sensor	Combined sensor	
			DES	MON
Slope (mV. decade^−1^)	−30.11	27.70	−29.16	29.79
Intercept	345.93	−10.17	337.60	−119.73
Linearity range (M)	5.00 × 10^−5^ − 1.00 × 10^−2^	1.00 × 10^−5^ − 1.00 × 10^−2^	1.00 × 10^−5^ − 1.00 × 10^−2^	5.00 × 10^−6^ − 1.00 × 10^−2^
Correlation coefficient	0.9996	0.9997	0.9998	0.9992
LOD^a^ (*µ*M)	0.036	0.018	0.025	0.025
LOQ (*µ*M)	0.109	0.055	0.075	0.076
Working pH range	2.5–5	6–10	2.5–5	6–10
Response time (seconds)	13	17	20	21
Lifetime (days)	63	49	49	49
Accuracy^b^ (R%)	100.95	99.60	98.48	101.27
Repeatability^c^ (RSD%)	1.53	0.31	0.98	0.44
Intermediate precision^d^ (RSD%)	1.90	0.86	1.05	1.19

^a^.Lod 3.3 SD of intercept/slope, and LOQ = 10 ∗ SD/slope. ^b^Average of three determinations. ^c^Repeatability: the intraday precision (*n* = 3 × 3) and average of three concentrations (5 ∗ 10^−5^, 5 ∗ 10^−4^, and 5 ∗ 10^−3^ M) were repeated three times within the day. ^d^Intermediate precision: the interday precision (*n* = 3 × 3) and average of three concentrations (5 ∗ 10^−5^, 5 ∗ 10^−4^, and 5 ∗ 10^−3^ M) were repeated three times on two consecutive days.

**Table 2:** Effect of the coating solution composition % (*w*/*w*) of DES- and MON-coated graphite sensors.

	Composition				Linearity (M)	Slope mv. decade^−1^	
	PVC%	DBP%	IP%				
DES.RNK sensor	47.75	47.75	5		5 × 10^−4^ − 1 × 10^−2^	−24.8	
	45	45	10		5 × 10^−5^ − 1 × 10^−2^	−25.6	
	60	30	10		5 × 10^−5^ − 1 × 10^−2^	−26.1	
	30^*∗*^	60	10		5 × 10^−5^ − 1 × 10^−2^	−30.1	
	40	40	20		5 × 10^−5^ − 1 × 10^−2^	−26.3	
	35	45	20		5 × 10^−5^ − 1 × 10^−2^	−26.2	
MON-Cd. sensor	47.5	47.5	5		5 × 10^−5^ − 1 × 10^−2^	23.8	
	45	45	10		1 × 10^−5^ − 1 × 10^−2^	24.9	
	60	30	10		1 × 10^−5^ − 1 × 10^−2^	26.1	
	30^*∗*^	60	10		1 × 10^−5^ − 1 × 10^−2^	27.7	
	40	40	20		1 × 10^−5^ − 1 × 10^−2^	25.2	
	35	45	20		1 × 10^−5^ − 1 × 10^−2^	25.7	
Combined sensor	30	60	5	5	1 × 10^−4^ − 1 × 10^−2^	−23.12	24.23
	40	40	10	10	5 × 10^−5^ − 1 × 10^−2^	−27.56	28.50
	24^*∗*^	48	10	10	1 × 10^−5^ − 1 × 10^−2^	−29.16	29.79
	45	35	10	10	1 × 10^−5^ − 1 × 10^−2^	−28.76	29.42

^∗^Best percentages of the components which gave the best sensor's characteristics.

**Table 3:** Selectivity coefficients of DES- and MON-coated graphite sensors.

Interfering	DES.RNK sensor	MON.Cd. sensor	Combined sensor	
			DES	MON
	K_DES, B_	K_MON, B_	K_DES, B_	K_MON, B_
K^+^	3.7 × 10^−3^	1.6 × 10^−3^	3.3 × 10^−3^	1.4 × 10^−3^
Na^+^	4.4 × 10^−3^	3.1 × 10^−3^	4.1 × 10^−3^	3.2 × 10^−3^
NH4 ^+^	2.6 × 10^−3^	7.8 × 10^−3^	2.2 × 10^−3^	7.5 × 10^−3^
Ca^2+^	1.6 × 10^−3^	3.9 × 10^−3^	1.3 × 10^−3^	3.6 × 10^−3^
Mg^2+^	1.8 × 10^−3^	8.1 × 10^−3^	1.6 × 10^−3^	8.3 × 10^−3^
Mn^2+^	2.1 × 10^−3^	8.6 × 10^−3^	2.2 × 10^−3^	8.7 × 10^−3^
Cu^2+^	4.5 × 10^−3^	7.4 × 10^−3^	4.4 × 10^−3^	7.6 × 10^−3^
Fe^2+^	5.1 × 10^−3^	6.8 × 10^−3^	5.3 × 10^−3^	6.6 × 10^−3^
Cd^2+^	3.9 × 10^−3^	6.5 × 10^−2^	4.1 × 10^−3^	6.2 × 10^−2^
Avicel	1.9 × 10^−3^	7.1 × 10^−3^	1.5 × 10^−3^	7.3 × 10^−3^
Mg stearate	2.3 × 10^−3^	4.5 × 10^−3^	1.9 × 10^−3^	4.3 × 10^−3^
Dextrose	3.3 × 10^−3^	6.2 × 10^−3^	3.1 × 10^−3^	5.8 × 10^−3^
Desloratadine	—	1.1 × 10^−2^	—	1.3 × 10^−2^
Montelukast	1.5 × 10^−2^	—	1.2 × 10^−2^	—

**Table 4:** Determination of DES and MON in laboratory prepared mixtures by the proposed sensors.

Ratio		Recovery %			
DES	MON	DES.RNK sensor	MON.Cd. sensor	Combined sensor	
				DES	MON
1	1	100.65	100.98	100.54	100.92
1	2	99.93	100.56	99.89	99.75
1	5	98.23	100.23	98.11	100.05
2	1	100.18	99.34	100.08	99.22
5	1	100.87	98.92	100.76	100.79
Mean ± SD		99.97 ± 1.04	100.01 ± 0.86	99.71 ± 1.30	100.15 ± 0.71

**Table 5:** Determination of DES and MON in pharmaceutical preparations by the constructed sensors and reported methods.

Commercial name	Composition	Amount found, mg^a^	R% ± SD	*t* value^b^	*F* value^c^
Sensor 1 DES.RNK					
Aerius	Desloratadine, 5 mg	4.980	99.60 ± 1.80	1.77	1.41
Azmalir	Montelukast sodium, 10 mg	—	—	—	—
Desolate-M	Desloratadine, 5 mg	4.993	99.87 ± 1.22	0.89	1.55
	Montelukast sodium, 10 mg	—	—	—	—
Sensor 2 MON.Cd.					
Aerius	Desloratadine, 5 mg	—	—	—	—
Azmalir	Montelukast sodium, 10 mg	9.990	99.90 ± 0.85	0.30	2.34
Desolate-M	Desloratadine, 5 mg	—	—	—	—
	Montelukast sodium, 10 mg	9.916	99.16 ± 0.61	.0.92	1.00
Sensor 3 combined sensor					
Aerius	Desloratadine, 5 mg	4.990	99.80 ± 1.60	1.04	1.11
Azmalir	Montelukast sodium, 10 mg	9.960	99.60 ± 1.15	0.60	1.27
Desolate-M	Desloratadine, 5 mg	5.020	100.40 ± 1.05	0.76	0.52
	Montelukast sodium, 10 mg	9.920	99.20 ± 0.92	0.83	2.27

^a^Average of 3 replicates; ^b^*t* critical 4.302 (0.05); ^c^*f* critical 19 (0.05); *n* = 3.

## Data Availability

The datasets used and analysed during the current study are available from the corresponding author upon request.

## Abbreviations

DES: Desloratadine
MON: Montelukast sodium
RNK: Ammonium reineckate
Cd: Cadmium chloride
PVC: Polyvinyl chloride
DBP: Dibutyl phthalate
THF: Tetrahydrofuran
ICH: The International Council for Harmonization of Technical Requirements for Pharmaceuticals for Human Use.
